# Cumulative summation analysis of learning curve for endoscopic endonasal transsphenoidal resection of craniopharyngiomas

**DOI:** 10.3389/fsurg.2024.1146957

**Published:** 2024-02-28

**Authors:** Jiye Ye, Ruiting Yang, Jie Wu, Chunming Xu, Tao Hong

**Affiliations:** ^1^ Department of Neurosurgery, The First Affiliated Hospital, Jiangxi Medical College, Nanchang University, Nanchang, Jiangxi, China; ^2^ Department of Neurosurgery, Hubei Provincial Hospital of Integrated Traditional Chinese and Western Medicine, Wuhan, Hubei, China

**Keywords:** craniopharyngioma (CP), endoscopic endonasal transsphenoidal resection, cumulative summation analysis, learning curve, neurosurgery

## Abstract

**Background:**

To evaluate the cumulative summation (CUSUM) analysis of the learning curve for Endoscopic Endonasal Transsphenoidal resection of craniopharyngioma (EETC).

**Methods:**

Retrospectively analyzed the clinical data of 113 patients who underwent EETC by the same neurosurgery team of the first affiliated Hospital of Nanchang University from June 2012 to November 2020. The learning curve was created by the CUSUM method and analyzed, which was divided into two groups: the learning stage and stable stage based on the learning curve trend. The median operation time and minimum surgical case number was calculated and the operation time and postoperative complications were compared between the two groups.

**Results:**

The median operation time was 318 min. The best fitting curve equation was *y* = 227.72 + 49.06*x* + 0.14*x*^2 ^− 0.05*x*^3^, *R*^2^ = 0.949, (*p* < 0.001). The minimum number of surgical cases was 65. Between the two groups, the operation time decreased from 360.8 ± 106.4 min in the learning group to 281.6 ± 69.9 min in the stable group (*p* < 0.05). The incidence of postoperative complications (intracranial infection, cerebrospinal fluid rhinorrhea, and diabetes insipidus) was significantly reduced (*p* < 0.05).

**Conclusion:**

The CUSUM learning curve of craniopharyngioma resection via endoscope endonasal transsphenoidal approach could better describe the learning process for a neurosurgeon. The frequency of surgery could be a good factor for strengthening the learning effect and help to shorten the learning time. After 65 cases of EETC, the surgical skills can reach a stable stage, the operation time is obviously shortened, and the postoperative complications are significantly reduced.

## Introduction

Craniopharyngiomas are rare brain tumors located either in the sella turcica (containing the pituitary gland; intrasellar) or above the sella turcica (suprasellar) and treated primarily with surgery (1). Though Albert E. Halsted successfully performed the first transsphenoidal resection of a CP in 1909 (2). Endoscopic Endonasal Transsphenoidal resection of craniopharyngioma (EETC) is still difficult to operate and requires high technical skills and long training for Neurosurgeons to be proficient.

The concept of the learning curve was first identified in 1936 by TP Wright and aimed to study the relationship between cost, speed, and component production of airplanes (3). Afterward, learning curves are referred to in the context of education and training in medical fields and have been suggested that all surgeons should recognize them when undertaking a new procedure (4). The cumulative summation test for learning curve, has been developed to quantitative and individual assessment of the learning curve, which could better describe the progress of a surgeon in different stages of learning a new surgical technique and quantify his proficiency (5) and has been used in many medical fields like ultrasound, gastrectomy, pancreaticoduodenectomy, spinal surgery and robotic surgery, etc (6–9).

A few studies have been reported on learning curves of the endoscopic endonasal approach for craniopharyngioma removal (10–12). However, none of the studies have performed cumulative summation (CUSUM) analysis. We retrospectively analyzed the clinical data of EETC performed by the same neurosurgical team to explore the learning curve of EETC in the CUSUM method and assessed its application on the therapeutic effect and postoperative complications. It has important guiding significance for the development and promotion of EETC.

## Materials and methods

### Clinical data and definitions

This retrospective study was approved by the local ethics committee.The clinical data of 113 patients who underwent EETC by the same neurosurgical team in the first affiliated Hospital of Nanchang University from June 2012 to November 2020 were collected by the author via electronic medical record system.

Variables included as following: age, gender, tumor type/size, primary or recurrence, resection extent, prognosis and complication. Tumor type is based on the relationship between the pituitary stalk and the origin. Central type and peripheral type are defined by the research group as previously described in detail by Bin Tang, et al. (13). Tumor size is calculated according to the length, width, and height measured from MRI images. The calculation formula is A × B × C × π/6, A, B, and C represents the maximum diameters in each of the three dimensions. Resection extent was classified as follows: Gross-total resection (GTR) was defined as no residual enhanced lesion or residual calcification. Subtotal (STR) meant more than 80% of the tumor was resected. The treatment effect of diabetes insipidus was divided into four levels according to its status pre-operation and post-operation: cured (positive pre-operation and negative post-operation), effective (both negative), ineffective (both positive), and aggravated (negative pre-operation and positive post-operation).

Inclusion criteria: (1) no serious organic disease, good cardiopulmonary function, able to tolerate anesthesia and endoscopic endonasal transsphenoidal surgery; (2) preoperative examination and pathological results diagnosed as craniopharyngioma; (3) complete and reliable clinical case data. Exclusion criteria: (1) severe organic diseases, poor cardiopulmonary function, difficulty tolerating anesthesia and operation; (2) postoperative pathological diagnosis of non-craniopharyngioma. (3) the clinical case data are incomplete.

All operations were performed by the same neurosurgical team, and the operators and assistants were trained and certified in neurosurgery. The instrument nurse is a specialist nurse in neurosurgery. The operator, the first assistant, and the instrument nurse are relatively fixed. Surgeries were performed by two neurosurgeons: the main operator and the first assistant. The main operator had 30 years of conventional microscopic transsphenoidal resection of craniopharyngiomas experience and the first assistant had 10 years experience in neurosurgery microscope operation. Before using EETC method, all surgeons and the surgical team followed a learning course on cadavers. All cases were operated in the same hospital, no ENT surgeons involved and no other surgeries which could have influenced the learning curve were performed by the surgeons in the study period.

### Operation procedure

The patient was supine with the head fixed to the head frame, 15° upward and 10° left. All patients were registered with neuro-navigation. The nasal cavity, mouth, forehead and face were disinfected three times with iodine. A 2-surgeon, 4-handed technique with bi-nostril access proceeded. Follow these steps to complete the operation: Intranasal operation (excision of the right middle turbinate, preparation of pedicled nasal flap, the opening of anterior wall of the sphenoid sinus)—the sphenoid sinus stage(removal of sphenoid mucosa and grinding of anterior wall of sphenoid sinus, bottom wall, sellar floor and sellar tubercle, MOCR, sphenoid platform and even slope bone as required by the bone window)—incision of dura and arachnoid to fully expose the tumor—resection of tumor—reconstruction of skull base—Nasal tamponade and hemostasis. The time was recorded as operation time for later analysis. All patients received preoperative antibiotics within 1 h before surgery and continued for 24 h postoperatively to prevent intracranial infection.

### Learning curve in CUSUM method

The learning curve represents the process of mastering a new method. It is usually measured by the number of surgical cases required for beginners’ surgical techniques to be relatively stable (14, 15). Since the CUSUM analysis method was applied to the study of the learning curve in the medical field in 1974 (16), the method has been more and more widely used in the study of the surgical surgery learning curve (17–22).

To detect the trend of operation time (operation time, OT), the CUSUM analysis method was used. CUSUM1 refers to the difference between the first operation time (OT1) and the mean operation time (OTmean), CUSUM1 = OT1-OTmean. CUSUM2 refers to the difference between the second operation time (OT2) and the average operation time OTmean plus CUSUM1, CUSUM2 = (OT2-OTmean) + CUSUM1, and so on. The specific formula is as follows: CUSUMn = (OTn-OTmean) + CUSUM (*n*-1), *n* represents the number of operation cases, OTn represents the nth operation time, and OTmean represents the average operation time of all operations.

### Curve regression

The CUSUM values of all surgical cases were counted by SPSS27.0 software, the CUSUM scatter diagram was drawn and the curve was fitted. The goodness of fit is judged by the fitting coefficient *R*^2^. The closer the *R*^2^ value is, the better the fitting degree of the fitting curve is. Linear, quadratic and cubic curves were fitted respectively, and the largest *R*^2^ was taken as the best fitting curve, and the curve fitting was significant when *p* < 0.05. When the slope value of the best fitting curve changes from positive to negative, it means crossing the learning curve, that is, the number of surgical cases corresponding to the highest point of the CUSUM fitting curve is the minimum number of surgical cases needed to cross the learning curve. According to the trend of the CUSUM fitting curve, the critical point was determined, and the learning curve was divided into two stages: the learning stage and the stable stage. All cases were divided into groups A and B equal to the learning and stable stages. The general clinical data such as gender, age, tumor type, tumor size, resection extent, primary/recurrence, mortality, and perioperative clinical data such as operation time, interval time, and postoperative complications such as intracranial infection, rate of cerebrospinal fluid rhinorrhea and treatment effect of diabetes insipidus were compared between the two groups.

### Statistical analysis

SPSS27.0 software (IBM Corp.) was used for statistical analysis. Measurement data subject to normal distribution uses $\bar{x}\pm s$. Group comparison uses an Independent sample *t*-test, chi-square test, or Fisher exact probability test, and the Levene test is used for the homogeneity of variance test. A rank sum test of nonparametric one-way ordered data was used for rank data (Mann–Whitney *U* test). SPEARMAN rank correlation coefficient was used for correlation analysis and partial correlation analysis. Probability values were 2-sided and *p* < 0.05 means the difference is statistically significant.

## Results

### Composition of clinical data

There were 65 males and 48 females in 113 patients. The minimum age is 4 years old, the maximum age is 71 years old, and the average age is 38 years old. 45 cases were central type and 68 cases were peripheral type. 90 cases were initially diagnosed and 23 cases recurred. 98 cases were resected and 15 cases were subtotal resected. Cases died after the operation. 17 cases had an intracranial infection. Cerebrospinal fluid rhinorrhea occurred in 18 cases. The symptoms of diabetes insipidus were cured in 7 cases, effective in 38 cases, ineffective in 24 cases, and aggravated in 44 cases (Table 1).

**Table 1:** Composition of clinical data.

Variables	Patients (*n* = 113)
Age (year)	
Minimus	4
Max	71
Gender	
Male	65
Female	48
Tumor type	
Central	45
Peripheral	68
Primary/recurrence	
Primary	90
Recurrence	23
Resection extent	
GTR	98
STR	15
Prognosis	
Alive	108
Dead	5
Complication	
Intracranial infection	17
Cerebrospinal rhinorrhea	18
Diabetes insipidus	
Cured	7
Effective	38
Ineffective	24
Aggravated	44

### The relationship between operation time and case number

**Figure 1:**
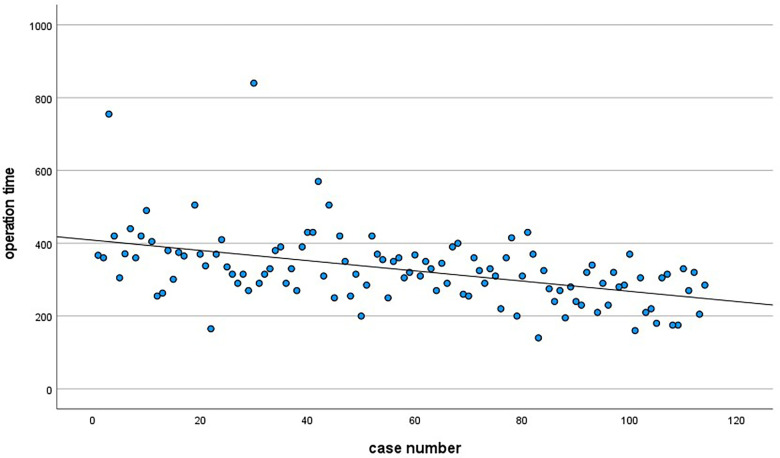
Relationship between operation time and case number of surgery.

All 113 patients successfully underwent EETC, and no intraoperative conversion to craniotomy was found. The longest operation time is 840 min, and the shortest is 140 min. The overall average operation time is 327.19 ± 9.44 min. With the increasing number of operations, the overall operation time is slowly decreasing. A scatter diagram of operation time and the case number is shown in Figure 1.

### CUSUM learning curve

**Figure 2:**
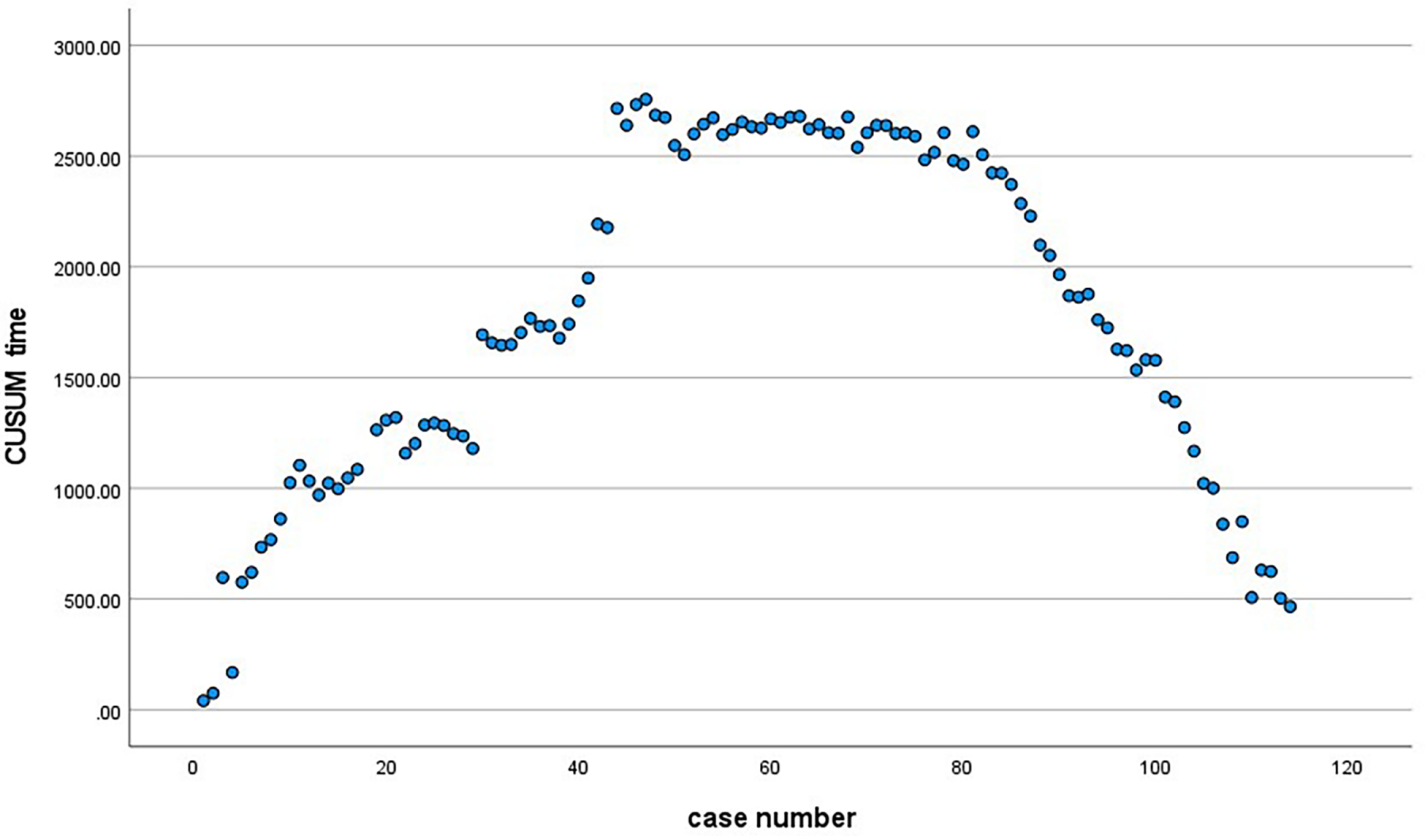
The scatter plots of the CUSUM time and case number.

**Figure 3:**
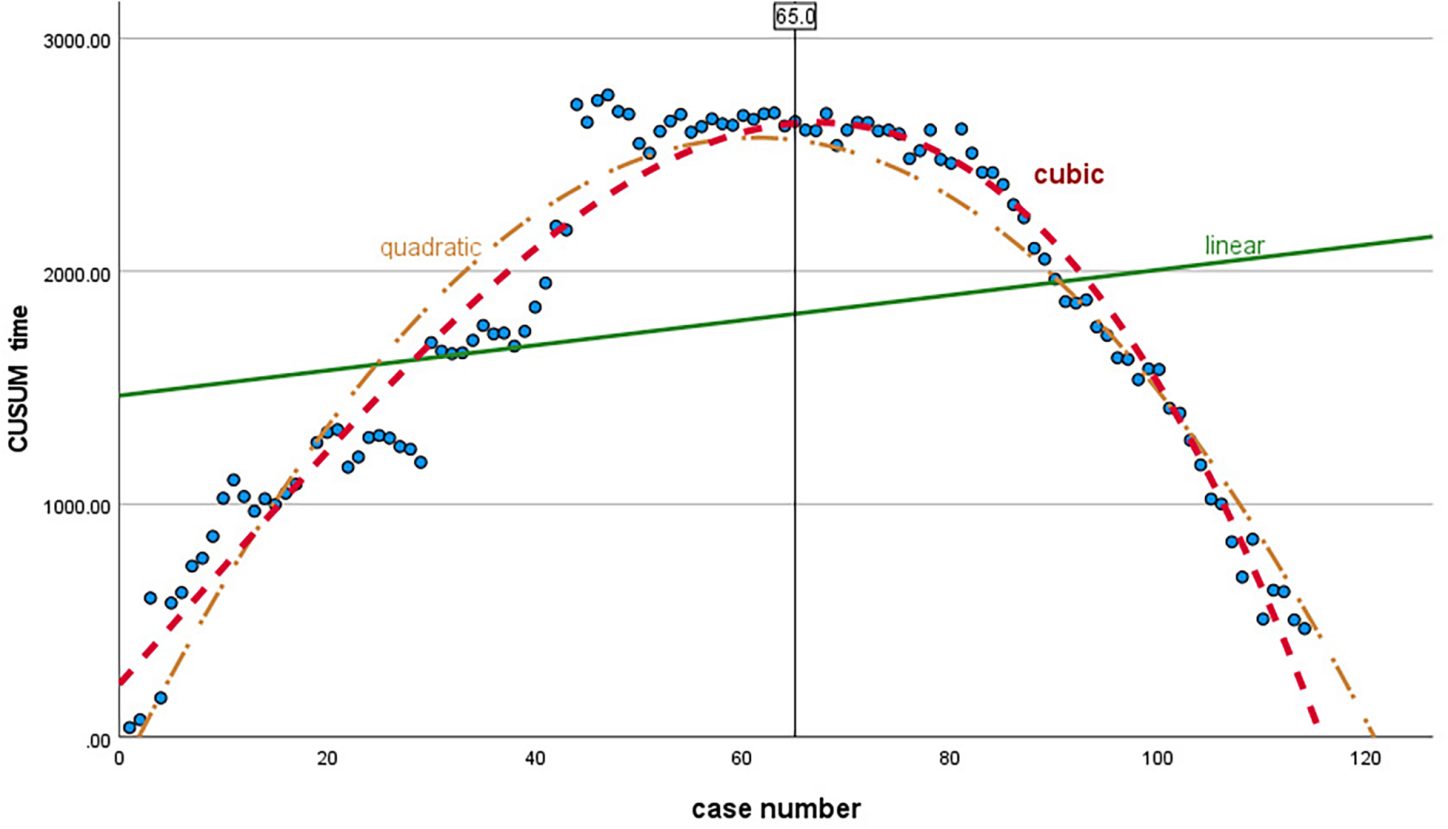
Curve model of regression: linear (green, *R*^2 ^= 0.054), quadratic (yellow, *R*^2 ^= 0.915), and cubic (red, *R*^2 ^= 0.949) regression. The best-fitting curve model is a cubic curve, *R*^2 ^= 0.949, *p* < 0.001, and the equation is *y* = 227.72 + 49.06*x* + 0.14*x*^2 ^− 0.05*x*^3^.

Use SPSS27.0 software to make statistics on CUSUM values of all surgical cases, draw a CUSUM trend chart (Figure 2), and perform curve fitting. The best-fitting curve model is a cubic curve (Figure 3), and the best-fitting curve equation is *y* = 227.72 + 49.06*x* + 0.14*x*^2 ^− 0.05*x*^3^, *R*^2 ^= 0.949, *p < *0.001. The CUSUM curve reached its peak at 65 cases of operation, and then the slope changed from positive to negative. 65 cases were the minimum number of operations needed to cross the learning curve.

### Comparison and analysis of clinical data

Taking 65 cases as the critical point, the learning curve was divided into two stages: the learning stage and the stable stage, corresponding to group A and group B. There was no significant difference in age, gender, tumor type, tumor size, resection extent, primary/recurrence, and mortality between group A and group B (*p* > 0.05) (Table 2).

**Table 2:** Comparison of clinical data.

Variables	Group A (*n* = 65)	Group B (*n* = 48)	T or *χ*^2^	*P*-value
Age (year)	41.15 ± 16.75	34.23 ± 18.45	2.08^a^	0.053
Gender			0.022^b^	0.881
Male	37 (56.9%)	28 (58.3%)		
Female	28 (43.1%)	20 (41.7%)		
Tumor type			0.537^b^	0.464
Central	24 (36.9%)	21 (43.8%)		
Peripheral	41 (63.1%)	27 (56.3%)		
Tumor size (cm^3^)	12.35 ± 16.317	12.19 ± 13.331	0.055^a^	0.956
Resection extent			0.592^b^	0.442
GTR	55 (84.6%)	43 (89.6%)		
STR	10 (15.4%)	5 (10.4%)		
Primary/recurrence			0.012^b^	0.913
Primary	52 (80.0%)	38 (79.2%)		
Recurrence	13 (20.0%)	10 (21.8%)		

^a^ *t*-test.

^b^ *χ*^2^-test.

### Comparison of perioperative data

There were significant differences between group A and group B in operation time, interval time, the rate of postoperative intracranial infection, and the incidence of postoperative cerebrospinal fluid rhinorrhea. In group B, the operation time was significantly shortened, and the incidence of intracranial infection and cerebrospinal fluid rhinorrhea was significantly reduced (*p* < 0.05). There was no significant difference between the two groups in the treatment effect of diabetes insipidus after the operation (*p* > 0.05) (Table 3).

**Table 3:** Comparison of perioperative data.

Variables	Group A (*n* = 65)	Group B (*n* = 48)	*t*/*χ*^2^/U	*P* value
Operation time (minutes)	360.82 ± 106.459	281.67 ± 69.927	4.483^a^	<0.001
Interval time (days)	29.48 ± 26.740	18.77 ± 25.326	2.156^a^	0.034
Intracranial infection	14 (21.5%)	3 (6.3%)	5.049^b^	0.025
Cerebrospinal fluid rhinorrhea	16 (24.6%)	4 (8.3%)	5.025^b^	0.025
Mortality	3 (4.6%)	2 (4.2%)	0.013^b^	0.909
Diabetes insipidus mean rank	52.41	63.22	1261.500^c^	0.067
Cured	4	3		
Effective	29	9		
Ineffective	9	15		
Aggravated	23	21		

Interval time, the time between adjacent operations.

^a^ *t*-test.

^b^ *χ*^2^-test.

^c^ Mann–Whitney *U* test.

### Correlation analysis

To explore the relevant factors affecting the operation time, the parameters of the operation time, number of cases, tumor size, and interval time were by normal distribution through the normality test. Pearson correlation coefficient was used for correlation analysis.

**Figure 4:**
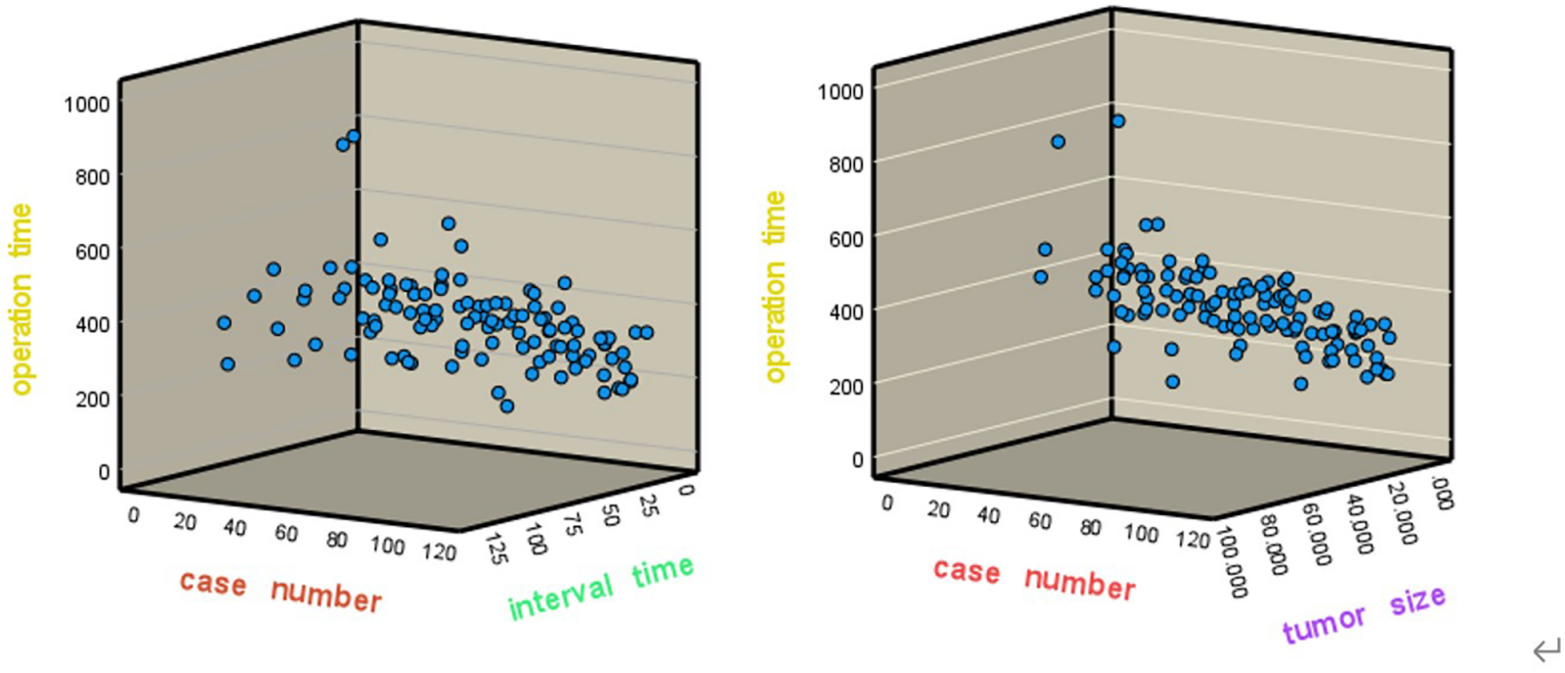
The scatter plots of the operation time, case number and interval time(left), and tumor size (right).

(1) There was a negative correlation between the operation time and case numbers, the correlation coefficient was −0.462, which was statistically significant (*p* < 0.001).
(2) There was a positive correlation between operation time and the tumor size, the correlation coefficient was 0.334, which was statistically significant (*p* < 0.001);
(3) There was a positive correlation between the operation time and the interval time, with a correlation coefficient of 0.117, which was not statistically significant (*p* > 0.05).
(4) The scatter plots of the operation time, operation cases and operation interval and tumor size are respectively shown in Figure 4. The results of the partial correlation analysis are shown in Table 4. On the premise of controlling the tumor size and interval time, the partial correlation analysis between the case number and the operation time still showed a negative correlation, with a coefficient of −0.477, which was statistically significant (*p* < 0.001). This means on the same condition of tumor size and interval time, the operation time will still be gradually shortened with the increase of surgery case number.

**Table 4:** (Partial) Correlation analysis result.

Controlled variable(s)	Variable		Case number	Tumor size	Interval time
	Operation time	Pearson coefficient	−0.462	0.334	0.117
		*P* value	0	0	0.218
Tumor size & Interval time	Operation time	Pearson coefficient	−0.477		
		*P* value	0		

## Discussion

Endoscopic endonasal transsphenoidal resection of craniopharyngioma (EETC) has created a minimally invasive neurosurgery for craniopharyngioma resection (23). Compared with traditional transsphenoidal or craniotomy microsurgery for craniopharyngioma, EETC takes advantage of three-dimensional imaging, high magnification, sufficient light source, clear vision, flexible steering, close observation, and fine manipulation of the skull base, which has been proven to be safe and effective (24, 25). With development and technology promotion, TTEC will be more and more widely used in clinical operations.

In this study, the CUSUM method was fist time used to draw the learning curve of EETC. The best curve was obtained through curve fitting analysis methods, which can more objectively reflect the learning process. The results of this study indicate that the learning curve of EETC can be divided into a learning stage and a stable stage. According to the best fitting CUSUM curve, 1–65 cases represent the learning stage, reflecting the process of operators’ familiarity with endoscopic microsurgery, and constantly exploring and cultivating mature operation techniques. 65–113 cases represent the stable stage. At this stage, the neurosurgeons have been able to master the key points of EETC, and have formed mature operation habits and technical skills.

The two groups corresponding to the two stages are comparable based on the statistical result that there is no significant difference in the composition of the two groups such as age, gender, tumor type, tumor size, resection extent, and primary/recurrence (*p* > 0.05); There are significant differences in operation time, the incidence of intracranial infection, cerebrospinal fluid rhinorrhea, and the interval time between operations (*p* < 0.05). It indicates that after a period of endoscopic surgery practice, the skills level and treatment effect of doctors are constantly improving, which can effectively shorten the operation time; It can significantly reduce the incidence of intracranial infection and cerebrospinal fluid rhinorrhea after the operation. Cerebrospinal fluid rhinorrhea, intracranial infection, and diabetes insipidus are common complications after craniopharyngioma surgery (26, 27). Cerebrospinal fluid rhinorrhea and intracranial infection are directly and mostly related to skull base reconstruction (28, 29). The results of this study showed that the incidence of cerebrospinal fluid rhinorrhea and intracranial infection decreased significantly at the stable stage. It shows that systematic training and operations can effectively control and reduce postoperative complications. Diabetes insipidus is a disorder characterized by the excretion of large amounts of hypotonic urine. It is the most common postoperative complication of craniopharyngioma resection (30). It has been reported that the highest incidence is 62% in craniopharyngioma resection and is highly relative to intraoperative cerebrospinal fluid (CSF) leak and intact function of the hypothalamus or pituitary gland (31, 32). However, there was no significant difference between the two stages in the treatment effect of diabetes insipidus before and after the operation. This result may be limited by the small case number, same as no significant difference in mortality, while it also could be contributed to the protection of the hypothalamus and pituitary during the operations. To a certain extent, it could play a positive role in effectively eliminating the psychological burden of the doctor caused by fear of insufficient clinical experience. Neurosurgeons can be encouraged to increase the surgical case number at ease status and ensure the maximum benefit for patients and doctors.

The results of correlation analysis in this study showed that the operation time was negatively correlated with the number of cases, indicating that the operation time would be gradually shortened with the increase in the number of cases. The positive correlation between the operation time and the tumor size means that the larger the tumor, the wider the scope of surgery involved, and the longer time the operation takes. The positive correlation between the operation time and the interval time means that a large number of operations in a short time would be helpful to improve surgical skills and shorten the operation time. On the contrary, if the interval time becomes longer, time to master skills of EETC will be delayed, and the operation time will become longer. The results of this study analysis are not statistically significant. It may be related to the small case number. The sample size needs to be further expanded for comparative study in the future.

Compared to traditional learning curve study in endoscopic endonasal resection of craniopharyngiomas (11, 12), there are no comparison between postoperative visual acuity and hormone levels in this research, such limitations could lead to bias, and this study was based on retrospective analysis of single center clinic, multicenter prospective studies are needed in the further research. Despite these shortcomings, this study is the first to use the CUSUM method to draw a learning curve of EETC, clearly and succinctly showing the growth process of neurosurgeons from beginners to proficient master. It has important reference value for the growth of neurosurgeons and the promotion of transsphenoidal endoscopic surgery for craniopharyngioma.

According to the learning curve analysis result, operation time can be shortened and the learning curve can be promoted by rich experience in craniotomy and microsurgery as important as frequency of operation and training intensity appropriately. Comprehensive knowledge of the sellar and parasellar anatomy is mandatory for the safe tumor removal with decreased morbidity and satisfactory oncologic results (33).

## Conclusions

The CUSUM learning curve of craniopharyngioma resection via endoscope endonasal transsphenoidal approach could better describe the learning process for a neurosurgeon. Proper frequency of surgeries could be good for strengthening learning effect and help to shorten the learning time. After 65 cases of EETC, the surgical skills can reach a stable stage of a learning curve, the operation time is obviously shortened, and the postoperative complications such as intracranial infection and cerebrospinal fluid rhinorrhea are significantly reduced.

## Data Availability

The original contributions presented in the study are included in the article/Supplementary Material, further inquiries can be directed to the corresponding author.
